# Indiana community health workers: challenges and opportunities for workforce development

**DOI:** 10.1186/s12913-022-07469-6

**Published:** 2022-01-27

**Authors:** Natalia M. Rodriguez, Yumary Ruiz, Ashley H. Meredith, Carlyn Kimiecik, Omolola A. Adeoye-Olatunde, Lynnet Francesca Kimera, Jasmine D. Gonzalvo

**Affiliations:** 1 College of Health and Human Sciences, Department of Public Health, Purdue University, 812 W. State Street, West Lafayette, IN 47907 USA; 2 College of Pharmacy, Department of Pharmacy Practice, Purdue University Center for Health Equity and Innovation, 640 Eskenazi Ave, Fifth Third Bank FOB, 3rd Floor, Indianapolis, IN 46202 USA

**Keywords:** Community health workers, Capacity building, Workforce, Indiana

## Abstract

**Background:**

An interest in, and the need for, Community Health Workers (CHWs) in the United States is growing exponentially. CHWs possess a unique ability to relate to and build trust with communities in order to improve clinical outcomes, while building individual and community capacity. Given their critical role in addressing social determinants of health, expanding the CHW workforce is crucial. However, creating CHW jobs, facilitating training and certification, and establishing sustainable financing models to support this workforce has been challenging.

**Methods:**

A mixed-methods study consisting of an online survey and focus group discussions assessed the strengths, practices, and challenges to CHW workforce sustainability and expansion in the state of Indiana, including perspectives from both CHWs and employers.

**Results:**

Across 8 topics, mixed data analysis revealed 28 findings that were both complementary and unique across focus group and survey results. Results highlighted CHW skills and attributes, illustrated the recruitment and hiring process, and provided insight into measuring outcomes and outputs. Findings also indicated a need to build position validation, professional development, and billing and reimbursement capacity.

**Conclusion:**

Building and sustaining the CHW workforce will require creating an evidence base of roles and impact, increasing awareness of existing reimbursement mechanisms, and sharing best practices across employer organizations to promote optimal recruitment, training, supervision, career development, and funding strategies.

## Background

As frontline public health workers, community health workers (CHWs) are trusted community members who have a close understanding of the community they serve [1]. This position of trust enables CHWs to serve as an essential link between health and social services and communities earning lower incomes who have been historically marginalized to facilitate access and improve the quality and cultural competence of service delivery [2]. CHWs improve clinical outcomes and build individual and community capacity by increasing health knowledge and self-sufficiency through a range of activities, such as outreach, community education, informal counseling, social support, advocacy, and community-based research and evaluation [2]. Kangovi and colleagues [3] reported a 28% reduction in hospitalizations following a CHW intervention in a population earning lower incomes with multiple chronic conditions. Ingram et al. [4] found CHWs significantly improved clinical markers (i.e., glycosylated hemoglobin, body mass index, and total cholesterol) of people living with chronic diseases. Furthermore, CHWs have been shown to save state Medicaid programs $4200 per beneficiary [5], and if scaled to even a quarter of United States (US) Medicaid beneficiaries, studies show CHWs would save taxpayers $78 billion annually [5].

Given CHWs’ critical role in addressing social determinants of health, including overcoming barriers to employment and financial self-sufficiency, expanding the CHW workforce is a powerful way to catalyze economic self-sufficiency in neighborhoods with incomes below the federal poverty levels. However, creating CHW jobs, facilitating training and certification, and establishing mechanisms to sustain this growing workforce has been challenging. Recently, Cacal [6] found that only 21 states in the US had legislative bills addressing CHWs and only 15 states had formalized definitions of CHWs. Establishing standardized definitions of CHWs is vital to reaching a consensus regarding CHW training, certification, and workforce development. Many states have modified the American Public Health Association (APHA) definition to enhance relevance in their communities (e.g., use of the word promotora [a lay health worker that works in a Spanish-speaking community]), which contributes to lack of clarity regarding CHW roles and titles [6]. In addition to a lack of a standard definition, there is no national consensus on CHW certification. A National Academy for State Health Policy (NASHP) survey reported only 17 states have certification pathways for CHWs. Certification is often voluntary but sometimes necessary for Medicare reimbursement [7].

In Indiana, sustaining CHW positions has been challenging due to limited funding and lack of infrastructure that fosters workforce growth and expansion. This has been due in part to poor awareness of CHWs and their roles, cost prohibitive training and certification opportunities for populations earning low incomes, and an underutilized and complex reimbursement system through Indiana Health Coverage Programs that fails to include a full range of CHW services. Despite these challenges, the employment of CHWs in Indiana is projected to grow at a rate of 14% from 2018 to 2028. With approximately 220 CHW job openings annually, this demonstrates a much faster rate of growth than the average for all occupations [8] and is associated with average annual earnings of $38,050 [9]. CHW certification in Indiana is open to anyone aged > 18 years with a high school diploma or equivalent, creating a promising employment pathway for young people and those without higher education, particularly for groups that have been marginalized [10, 11]. To promote the unification of CHWs, the Indiana Community Health Worker Association (INCHWA) has developed a framework through which Indiana CHWs may become certified. Working with the Indiana Department of Health, INCHWA approves “training vendors” certified by the state to train and produce Medicaid reimbursable certified community health workers. While certification is not a requirement to practice as a CHW in Indiana, it is required to receive reimbursement from Medicaid.

CHWDI was created in 2020 in recognition of the immensely important role and expected growth of CHWs, and to address CHW workforce development challenges in Indiana. Dedicated to health equity, CHWDI’s mission is to expand the Indiana CHW workforce to increase access to health and social services for under-resourced communities while also increasing employment opportunities for community members. Thus, to inform CHWDI’s statewide initiatives in Indiana, the objectives of this study were to conduct a statewide needs assessment to 1) characterize the current CHW workforce landscape, 2) describe CHWs’ experiences with training, certification, scopes of work, job security, career development opportunities, and community priorities, and 3) explore employers experiences in hiring, training, and supervising CHWs, current challenges and potential areas of needed training and development activities, and funding mechanisms to sustain or expand CHW employment opportunities.

## Methods

Utilizing a concurrent, multimethod approach [12], all aspects of this study, including the initial design, were informed though diverse stakeholder engagement. Specifically, in collaboration with INCHWA, a steering committee was established which included 25 members from public health and social service government agencies, academic institutions, and other key community stakeholders. The steering committee was convened to provide essential insight that will continuously inform the implementation, evaluation, and long-term sustainability of the institute’s efforts. This study was approved by the Purdue University Institutional Review Board (IRB; protocol IRB 2020–1468) and the review deemed this study exempt. Informed consent was obtained verbally by all participants prior to commencing research activities as approved by Purdue University’s IRB.

### Data collection instrument development

The research team and steering committee collaborated to identify objectives pertaining to data collection instrument development (i.e., survey, focus group questionnaire). Both quantitative and qualitative data collection instruments were framed by 13 pre-defined overarching topics (see Appendix).

### Quantitative instrument development

An initial survey was created by the research team, with feedback from the steering committee and INCHWA to ensure appropriate content and consistent language leading to minor modifications (e.g., providing additional response options and language clarity for intended audience and adding questions for additional contact-related information from participants) and four items added. The final 59-item survey sought to establish a demographic profile of Indiana CHWs, identifying CHW job titles, roles, employment sectors, and activities, as well as to capture CHW certification and training prevalence, and collect demographic information of communities and populations that Indiana CHWs serve.

### Qualitative instrument development

Concurrent with survey development, focus group data collection instruments followed a similar process as that of the survey. With the goals of receiving CHW and employer input related to the CHW workforce and gaining a greater understanding of strengths and challenges of the workforce and CHW roles, two semi-structured focus group guides (Appendix) were developed. Specifically, one guide was developed for CHWs and a separate guide for employers of CHWs.

### Quantitative data collection and analysis

In November 2020, an online survey of Indiana CHWs was conducted through use of an anonymous Qualtrics survey link distributed through INCHWA. Respondents could forward the link to additional CHWs. The survey remained open for 4 weeks. Only CHW respondents that indicated that they were based in Indiana were included in data analysis. Additionally, to eliminate potentially duplicative and fraudulent responses, researchers excluded responses from identical internet protocol (IP) addresses and email stems, that took 5 minutes or less to complete (minimum survey completion time of 10 min based on the estimated time to complete the survey), and that were less than 50% complete. SPSS software (Version 26) was used to descriptively analyze data (i.e., frequencies, counts). All eligible survey participants received a $10 e-gift card.

### Qualitative data collection and analysis

Focus group participants were recruited through INCHWA’s mailing lists, with all interested participants able to engage via Zoom videoconferencing software. Focus groups were facilitated by members of the research team over a four-week period in November–December 2020, with each focus group lasting between 75 and 105 min. All participants received a $45 e-gift card as compensation for their time. All focus groups were recorded verbatim and transcribed using Otter.ai software. Research assistants completed a quality check of each transcript to ensure accuracy prior to data analysis.

NVivo 12, a qualitative software, [13] was used to deductively and inductively analyze data to identify emergent topics [14, 15]. First, using the focus group guide a codebook with 13 pre-defined topics was developed and deductive coding was carried out. Next, an inductive analysis was conducted to further refine the pre-defined topics as well as add new topics to the qualitative codebook [15, 16]. Throughout the deductive and inductive analysis, coding consisted of identifying direct quotes that represented each topic and organizing these quotes, or raw data, into findings by topic while succinctly combining quotes into broader topics [6, 14]. Researchers met to review and discuss findings and any discrepancies were resolved through discussion [17].

### Mixed data analysis

To identify areas of concordance among data sources, survey and focus group data were triangulated using a data consolidation analytic approach [18, 19]. This approach blends qualitative and quantitative data to investigate a phenomenon, thus corroborating internal validity of findings [20]. Microsoft Word and Nvivo 12® [13] software were used to compare and consolidate findings via cross-tabulation of quantitative and qualitative findings by each of the pre-defined and inductively derived topics.

## Results

A total of 1804 survey responses were received with 1156 responses excluded due to not meeting inclusion criteria (see Methods section). A total of 648 responses were eligible for analysis. See Table 1 for demographics of survey respondents. Four total focus groups were conducted with participants representing 25 different organizations throughout Indiana. Two focus groups were conducted with CHWs (*N* = 7 and *N* = 7) and two with employers (*N* = 9 and *N* = 7). Across 8 topics, mixed data analysis of survey results and focus groups revealed 28 findings (Table 2). Overall, 6 (21%) findings were complementary and supported by both quantitative and qualitative data sets, 20 (71%) findings were unique to focus group results, and 2 (7%) findings were unique to survey results. There was no observed divergence of results between the two data sources (Table 2). The 8 topics are detailed below with both quantitative (survey) and qualitative (focus groups) results presented within the respected topic.

**Table 1:** Survey Participant Demographics

Variable	No. (%)
**Sex (*N*** **= 648)**	
Male	364 (56)
Female	280 (43)
Did not respond	4 (1)
**Race/Ethnicity (N = 648)**	
White	439 (68)*
Black or African American	61 (9)
Asian	19 (3)
Hispanic or Latino	29 (5)
Native Hawaiian or Pacific Islander	4 (1)
Two or More Races	89 (14)
**Education Level (N = 648)**	
Less than high school	11 (2)
High school or GED equivalent	92 (14)
Technical school/Some college or associates degree	288 (44)
Bachelor degree	157 (24)
Graduate degree or higher	92 (14)
Did not respond	8 (1)
**Currently a certified CHW in Indiana (N = 648)**	
Yes	526 (81)
No	65 (10)
No, but interested	29 (5)
Waiting for training certification, or testing results	10 (2)
Unsure	17 (3)
Did not respond	1 (0.2)
**Current/past employer paid for CHW certification (n = 526)**	
Yes	492 (94)
No	34 (6)
**Obtained CHW certification in Indiana (*n*** **= 526)**	
Yes	517 (98)
No	9 (2)
**Provide CHW services as paid staff or volunteer (N = 648)**	
Paid Staff	516 (79)
Volunteer	128 (20)
Did not respond	4 (1)
**Current hourly CHW wage (N = 648)**	
More than zero and up to $7.25	135 (21)
More than $7.25 and up to $14	247 (38)
More than $14 and up to $18	187 (29)
More than $18	56 (9)
0 (unpaid)	22 (3)
Did not respond	1 (0.2)
**Provide CHW services full-time or part-time (N = 648)**	
Full-time	561 (87)
Part-time	78 (12)
Did not respond	9 (1)
**Number of part-time hours per week (*n*** **= 78)**	
10 h or less	18 (23)
11–20 h	40 (51)
20 h or more	15 (19)
Did not respond	5 (7)
**Length serving at current organization (N = 648)**	
Less than a year	39 (6)
1–5 years	316 (48)
6–10 years	248 (38)
11–15 years	34 (5)
16–20 years	5 (1)
Over 20 years	4 (1)
Did not respond	2 (1)

**Table 2:** Triangulation of survey and interview findings by each of the 8 topics. Each finding is accompanied by an explanation of findings. Additionally, focus group and unique survey findings are accompanied by supporting quotations and survey results, respectively. The right column indicates whether survey and focus group findings were complementary, divergent, or unique. No findings were divergent

Findings by topic	Explanation of findings	Representative Quotations from Focus Groups and Survey Results	Comparison of Survey and Focus Group Results
**Topic 1: Who are Community Health Workers?**			
1. CHWs have diverse backgrounds	Many CHWs reported being multilingual, with many having international backgrounds, and receiving a variety of education beyond a high school degree equivalent.	See Table 1.	**Unique:** Survey
2. CHWs are known by many titles and represent various employment settings	CHW respondents reported on their existing job titles, employment sectors and settings, and health-related topics and services they provide to their clients, patients, and communities.	Survey results revealed 11 job titles held by CHWs (see Table 3 for CHW job titles) in addition to CHWs representing XX employment sectors and setting (see Table 4). Health-related topics covered by CHWs (see Table 6), include the most common focus area, chronic disease management (13%). Specific chronic diseases that CHWs focus on include high blood pressure (7%), obesity (5%), diabetes (3%), asthma (2%), kidney disease (2%), COPD (1%), Alzheimer’s (1%), arthritis (1%), and high cholesterol (< 1%).	**Unique:** Survey
3. CHW clients face a myriad of challenges	Challenges that CHW clients and the communities they serve face include food insecurity, unstable housing and homelessness, high medication costs, substance use, domestic violence, lack of accessible and affordable daycare, and undocumented status.	“And the biggest thing of course…is food resources and food deserts. That’s a big issue, at least here in my territory” —CHW “…in the community-based organizations in the community where I [work] I need housing, I need protection and the domestic violence situation, I need access to food. And those are things that really contribute to the health disparities that we’re seeing.”—EMP	**Complementary:** Survey & Focus Groups (CHW & EMP)
**Topic 2: Community Health Worker Demographic Attributes, Qualities, Skills, and Roles (23)**			
4. CHWs are highly motivated	CHWs indicated having a strong desire to help their communities and to work with clients from diverse backgrounds. This finding aligns with C3 Core CHW Role 1: *Cultural Mediation Among Individuals, Communities, and Health and Social Service Systems* and Role 2: *Providing Culturally Appropriate Health Education and Information.*	“I decided to move to this field because it is more related to compassion and passion for people and [people] need help.”—CHW “I love the idea of being able to work with families and to work with not just trying to prevent but intervening, and also trying to address other areas of social determinants of health.” —CHW	**Unique:** Focus Groups (CHW)
5. CHWs possess a myriad of qualities to effectively develop trust between themselves and communities served	Several attributes contribute to CHWs trustworthiness including, patience, compassion and passion, inclusivity, dependable and consistent, creative, and flexible, adaptable, shared life experiences, ability to set healthy boundaries. This finding aligns with C3 Core CHW Qualities.	“I think it’s really important to be very open-minded and non-judgmental and having a very open demeanor.” —CHW “We’re just kind of helping people with their barriers. And the addiction groups, we’re using our own addiction history, if some of us have that, to help people understand that it’s going to be okay, and it can be okay. It can even be better than before you know, so we’re kind of like that hope.” —CHW “Life and lived experience is more valuable than the credentialing that would come otherwise in helping families and be able to identify with the needs in the communities.”—EMP	**Unique:** Focus Groups (CHWs & EMP)
6. CHWs’ have shared life experiences and are relatable to community and clients	Often CHWs are from and/or live in the communities that they serve and can relate the clients they work with because of their own life and lived experiences. This finding aligns with C3 Core CHW Skill 11: *Knowledge base.*	“…having that knowledge base [previous life experiences] allowed me to really hone in on what was needed [in the community] and also to be able to convey information [to clients] in an easy to understand way.”—CHW “…I think you have to have that passion to work in this position. You come up against a lot of walls, a lot of barriers, and you’ve got to be very creative in how you address some of those issues. You don’t want to burn any bridges.”—EMP	**Unique:** Focus Groups (CHW & EMP)
7. CHWs are essential links between their clients, services, resources, and communities	CHWs and employers described CHW roles as bridges between their clients and the services they need and connect clients to medical and social services. This finding aligns with C3 Core CHW Skill 3: *Service Coordination and Navigation Skills* and Core CHW Role 3: *Care Coordination, Case Management, and System Navigation.*	“…intermediary between the clinicians and like real people…the community it’s a very important role.” —CHW “…six of our CHWs are on [that] diabetes project to see patients who are dying due to uncontrolled diabetes, and it helps to coordinate their care as well as linking them directly to community resources.”—EMP	**Complementary:** Survey & focus Groups (CHW & EMP)
8. CHWs address client needs’ and improve their lives through various actions and avenues	CHWs improve the lives of their clients by serving as advocates as well as developing and implementing programs to address health disparities in order to better client lives and wellness. This finding aligns with C3 Core CHW Role 5. *Advocating for Individuals and Communities*.	“Part of my job is to also help develop programs that address those needs that we [identify through] our needs assessments with the community.”—CHW “The CHWs that work here in the community are actually advocates…they do presentations on health disparities, blood pressure, diabetes…because most of our community members don’t’ have the proper documentation to actually go into a doctor’s office, or they don’t have the financial means…so they [CHWs] go into the community and talk to them about what is blood pressure, or how to use a blood pressure cuff. And they build rapport with them…guide them to the different resources that we have in the community.”—EMP	**Complementary:** Survey & Focus Groups (CHW & EMP)
**Topic 3: Community Health Worker Recruitment and Hiring**			
9. Hire CHWs to provide health education and bridge critical gaps to care and community resources	Employers emphasized that CHWs work with and advocate for individuals and communities at large to address identified needs that are not being met as well as providing programming and support that registered nurses and other medical professionals cannot disseminate.	“Our CHWs work both with individuals as well as neighborhoods. So addressing the individual’s specific needs, self, personal identified needs and goals, but then also the environment that allows for access…and so the importance of the neighborhood learning to advocate for their environmental needs, and to build a community is really helpful…that really doesn’t fall into case manager or social worker space.”—EMP “…identified those community resources that were reliable, that we knew were going to give good service, because we didn’t want our clients to be going someplace where they couldn’t receive, you know good care. So that’s a big part of the [CHW]work.”	**Unique:** Focus Groups (EMP)
10. Employer-identified facilitators of the hiring process	Facilitators of the CHW hiring process include, recruiting directly from client populations and include community partners, creating clear job descriptions with specific roles, provide full benefits to CHWs, have current CHWs present during interviews and onboarding, aim to hire internally, and include proper organizational leadership and relevant stakeholders during hiring process.	“…have the right people at the table at the beginning, even if [the CHW position is] not even approved yet. So that way we can plan, and other leaders are not blindsided to things that may impact their current workflow, or some of their staff.” —EMP “I feel really strongly that those CHWs do need to be from the community, known in the community as the natural or informal leader” —EMP	**Unique:** Focus Groups (EMP)
**Topic 4: Certification and Training**			
11. Benefits of certification	CHW certification provide legitimacy and validation and offers CHWs the opportunity to strengthen their overall skill-base.	“It was a good…certification…just to kind of let people know in the community I’ve been doing this for a while. I’m a CHW, and I’m focused on what’s going on in the community.” —CHW “The benefit to us for the community health worker certification, though, for our licensed Indiana navigators, that it just gives them another layer of competence. So as people contact us, you know, seemingly that they just need health coverage, but they might also need to be connected to the application for food stamps, or you know, other things. It’s just kind of this multi layered interaction with people and so that’s the benefit to us.”—EMP	**Unique:** Focus Groups (CHW & EMP)
12. Certification training programs and preferences	CHWs were introduced to certification and encouraged to become certified by their employers and felt that the in-person training was positive, provided opportunities to network, and was preferred.	“I did the weeklong in person, and it was a ton of information. I felt that it could have at least been a good additional 2 days. It was just so much.” —CHW “They [CHWs] really liked it…people always had good things to say about that [in-person] training.” —EMP	**Unique:** Focus Groups (CHW & EMP)
13. Certification vendors and guidance	CHWs reported what vendor they received their certification from while employers spoke of the guidance from the state regarding CHW certification.	“They’re [certifications] from various organizations and schools…Health Visions, IUPUI…I can’t think of them all.”—CHW “I think we just need the state to say, this is the training. And this translates to what you’ll be able to bill, without there being any question about that. And so I think the legislative piece, like that state piece, being in place, would be huge. And to know… this is the training that leads to this, that leads to you being able to bill through this, having that, and I know, we’re trying to piece all that together. But putting that to bed would be great… that would be a huge step.” —EMP	**Complementary:** Survey & Focus Groups (CHW & EMP)
14. Certification affordability	Certification was often paid for by the employer organizations, grant or funding agencies, or by scholarships and there are opportunities for certification cost reimbursement and discounts that are covered by the state. The many avenues for certification reimbursement and cost have led employers to feel confused.	“[Our employers paid for] the six of [to go] off site to another location and took our certification training with other people from other agencies. So someone came in and gave us all training, that 40 h training. We took it all in 1 week.”—CHW “I had heard that taking the class for just CHW [certification] was kind of expensive…it was even more money than just doing the whole CRS/CHW program. So I think that could deter some people from becoming CHWs” —EMP	**Complementary:** Survey & Focus Groups (CHW & EMP)
15. Continuing education	While continuing education is not required by the state of Indiana, CHWs expressed a desire to completed additional educational and training opportunities but had to seek these opportunities out on their own.	“Some annual conference or something that goes on every year, speakers are brought in and things like that…to learn from other CHWs about best practices would be very beneficial in person or on zoom at least.” —CHW “It [training] was on the front end. And then it was just me like ‘oh that looks good, I should learn about this.’ A lot of it is on us to stay trained. But then also like if we aren’t people that take initiative, we’re just missing out.”—CHW	**Complementary:** Survey & Focus Groups (CHW)
**Topic 5: Community Health Worker Job Satisfaction, Challenges, and Recommendations**			
16. Challenging aspects of the jobs	Challenges discussed by CHWs included a lack of understanding on who CHWs are and their positions, working in an undervalued and underpaid role, and a lack of resources to support their communities but feeling pressure to find resources anyway.	“Well, one of the problems we had before is, nobody had a clear definition of what a CHW was or does. And we’re still grouped into a lot of different things. We have people who do insurance, we have people who do education, we have folks who do goal setting, we’re still all over the place, so to speak. But it’s being defined more readily now. So people are starting to learn about it, because I don’t think my job really knew what a CHW did either.” —CHW “It kind of falls squarely on us when a patient has need for housing, like immediately, or has need for legal help, or whatever it may be. So I feel like it puts a lot of pressure on for us to kind of pull a rabbit out of the hat…”—CHW	**Unique:** Focus Groups (CHW)
17. Rewarding aspects of the job	CHWs enjoyed making a difference in the lives of and empowering their clients as well as being recognized by their team.	“Just knowing that you’ve helped somebody today, that what you’re doing literally could be changing somebody’s life. And that’s very powerful, very rewarding. And makes all of the hassles that you deal with at work on a daily basis, the end of the day, you go home, you know someone’s day, someone’s life is better, because you’ve stepped in.” —CHW “We sit down and have this action plan and kind of hold them accountable, it makes them feel good that they’re accomplishing it. And it makes me feel good that I gave them the tools and resources to make them feel competent in that.” —CHW	**Unique:** Focus Groups (CHW)
18. CHW recommendations	Recommendations to address identified job challenges were shared by CHWs. For example, expanding the workforce and receiving more CHWs, improving coordination and communication between service organizations and state agencies, demonstrating the importance of the workforce, and providing additional resources and trainings were offered to improve CHW positions.	“Some training on how to show your work because for grants, you have to show your work for your bosses, you have to show your work in order to impress them or show them the need for more of you.” —CHW “Be more visible in the community, so more people know what CHWs are, what we do.” —CHW	**Unique:** Focus Groups (CHW)
**Topic 6: Measuring Community Health Worker Impact**			
19. Importance of collecting and reporting data on CHW outcomes and outputs	Reporting CHW impacts justifies CHW position, demonstrates a return on investment, and makes the case to hire additional CHWs.	“I always wanted to show our outcomes. And I think that’s a good way to try and release yourself a little bit from grants to show how valuable you are to the organization. The bottom line is finances do count.” —CHW “You have to make the case mathematically. That is something that I think that statewide, we could do something about, like having a system where we could help CHWs show their math, show the work because administrators typically aren’t going to hire more people unless you can show them how it’s affected the budget…how many more patients were brought in, how many people actually showed up to their follow up visits…how many people got insurance instead of us putting them in the write off bucket…we go them insurance, and they are now insured patients instead of uninsured.” —EMP	**Unique:** Focus Groups (CHW & EMP)
20. Metrics and key performance indicators for employer-defined success	Employers described output metrics, outcome metrics, and metrics for CHWs who work in community development. Examples of these metrics and key performance indicators include number of enrolled patients and community outreach events, tracking referrals and program attendance, client-reported outcomes, and follow-up rates.	“We are outcome based as well. So we keep track of how many referrals we get each week and in our tracking system we don’t’ count until that person has received their medication or that person successfully received SNAP or HIP or Medicaid…”—EMP “And so now we’re at a better state because we’ve got with the Epic. We have what we call registries that can capture that information [A1C, ERA diabetic] behind the scenes, that looks at all that metrics, and all we have to do is plug in, like we enroll our patients within what we call episodes of care, which crack those patients who are in there. But we can pull reports that shows people within this episode what their data looks like compared to those who are not.”—EMP	**Unique:** Focus Groups (EMP)
21. Metrics and key performance indicators for CHW-defined success	CHWs identified establishing relationships with clients and community they serve, the amount and quality of resources provided to their clients and reaching programmatic benchmarks and enrollment goals as success measures.	“For me, I would define success as going into my job every day to the best of my ability, having a positive attitude, leaving any issues I may personally have the side and letting the customer or client be the focal point to try to find out whatever I can and fulfill what they need to try to help them because after all, they do appreciate what we do. So it’s important that we find the resources for them [clients] or make the connections for them. And once I do those things, I feel like I’m doing pretty good.” —CHW “For me, I would say using data, like our enrollment numbers every month and seeing if we had losses and membership of certain programs. And if you’ve seen an increase, what attributed to that increasing? So I will use enrollment data a lot to help gauge how I’m doing.” —CHW	**Unique:** Focus Groups (CHW)
22. Current and needed tools to track CHW outcomes and outputs	Several employers expressed the need for standardized tools to track metrics or key performance indicators of CHW impact. Some employers utilize electronic medical record-based registries, Recovery Inventory, Star, Strength and Risk Assessment, Child and Adolescent Needs and Strengths and Adult Needs and Strengths Assessment state tools, and Insight Vision	“One thing I notice is that when we were looking for evaluation tools for CHWs they’re not really there. If they are, I just haven’t found them because I thought that maybe there’d be some evidence-based tool out there that we could just pick up and then bring here and use. And I think we tend to do it more so on our own looking at these are the metrics we need to be measuring. But we’ve really looked at like different studies that are out there, but nothing has been usable that I’ve look for…I really would like to get out of grant world.” —EMP “So now it’s easily done by our EMR system, and a lot of it boils down to what are the health outcomes? Form a medical standpoint, how are these CHWs benefiting the patients and how’s their A1c improving…blood pressure control…health condition metrics that we have to monitor now, to be able to compare against our other cohorts who are not utilizing our service to say that this is the return on investment. I think we’ve gotten better with the EMR tool that we chose.” —EMP	**Unique:** Focus Groups (EMP)
**Topic 7: Supervision, Professional Development, and Peer support**			
23. Supervision	The potential for CHWs to “train-up” into a supervisory role and group and one-on-one supervision models were discussed. CHWs also described characteristics of an ideal supervisor as someone who has done the work of a CHW and is supportive.	“The support of the supervisor is very, very important. When they recognize what we are doing. And they realize that is really a good job. And, and it’s important. It’s, it makes a difference.” —CHW “I manage our CHWs, but I myself became a CHW, and I do the same role that they do, even though I supervise our entire department, I still do the referrals, I still do the community outreach, I do the home visits. So I know I am able to advocate for our advocates on what they need.” —EMP	**Unique:** Focus Groups (CHW & EMP)
24. Professional Development	CHWs expressed few opportunities for professional development and felt their positions did not have clear pathways for promotion. Desired advancement of their roles included becoming a supervisor, teaching other CHWs in programs beyond their current role, and creating new programs.	“So within the organization, there are not too many [promotions]. But if something comes open, and you’re interested in you can apply.” —CHW “I would like to advance at some point and either start a new program working with the same population or change a program. I’m not sure but something in an administrative role, at some point.” —CHW	**Unique:** Focus Groups (CHW)
25. Peer Support	INCHWA serves as the primary avenue for peer support. CHWs reported occasions participations in INCHWA functions such as email, huddles, and webinars. CHWs appreciated these INCHWA touchpoints to increase camaraderie and networking within the CHW community but expressed interest in an annual conference and were unclear of all INCHWA’s roles and responsibilities.	“I feel like they’re [INCHWA] advocating for CHWs from what I’ve seen, and for better pay, better representation, and more of a voice in the medical community. And I really applaud INCHWA for doing that [advocacy]. And making it seem legitimized as a profession.” —CHW “Maybe having an annual conference, I just feel like that just benefits us, everyone. People really enjoy that in the camaraderie…a yearly conference that we can go to and learn more things that we otherwise might not have the opportunity to.” —CHW	**Unique:** Focus Groups (CHW)
**Topic 8: Community Health Worker Funding and Reimbursement**			
26. CHW position funding	CHW position are supported by various sources of funding. Employers and CHWs agreed there is no guarantee of consistent job security for grand funded position which can lead to time-limited positions. Employers spoke about grant availability and the role funding plays in sustaining and extending the CHW workforce.	“We work for a not for profit. So things change there all the time, funding could go at any moment.” —CHW “We definitely need to know when different grant opportunities are available…I can look for grants, like take some time out to look for them…but with everything else, I have to do I just take that much more time to actually look for specific grants that fit my programs…because it’s never really been about not being able to write…it’s more about I didn’t’ get this grant and enough time to write it.” —EMP	**Unique:** Focus Groups (CHW & EMP)
27. Billing and reimbursement	CHWs and employers expressed confusion and lacked awareness of the details surrounding the logistics of CHW reimbursement. Participants encountered barriers to successful Medicaid reimbursement, including the inability to bill for every client and an array of CHW services that are not clearly defined within reimbursement language.	“…it’s a chicken and egg thing, if they would reimburse more, they would hire more community health workers, but a lot of places don’t see it as a valid investment and so they don’t bother with it. Can we at least have a decent reimbursement model of some kind?” —CHW “Because knowing that all these different levels of position exists, it’s hard to get a more concrete understanding of what’s a part of that CHW service definition.” —EMP	**Unique:** Focus Groups (CHW & EMP)
28. Employers offered suggestion for billing and reimbursement	Employers stated a need to expand opportunities to bill services provided by CHWs for reimbursement provided suggestions such as more effective collaboration between their organizations and the medical profession and Z code usage to support future reimbursement efforts.	“…if our doctors or providers would work closer together, and they would look through the curriculum with me, we could work as a partner, and I could educate, educate the group, and get reimbursed for his patients, because I’m helping his patients, they helped me.” —EMP “We use the Z codes and we’re starting to track those through the ICD-10 codes. And we’re gonna start working with those just because we’re trying to set it up is if we could bill. So we’re changing our HER and we’re able to attach with our service, what we’re doing for those people.” —EMP	**Unique:** Focus Groups (EMP)

### Topic 1: who are community health workers?

Quantitative analysis of survey results revealed that Indiana CHWs are highly diverse with 43 countries and 14 languages represented (Table 1). CHWs are employed under various titles (Table 3) and in various employment sectors and settings, including Social and Community Services (24%), County Health Departments (15%), and Private health sectors (13%) (Table 4). CHW services are provided throughout Indiana’s 92 counties and triangulated data supported existing literature [21, 22] that CHWs serve clients who face a myriad of economic, social, physical, and environmental challenges (Tables 2 and 5).

**Table 3:** Community Health Worker Job Titles

Job Title (N = 648)	No. (%)
Community health worker	208 (32)
Certified recovery specialist	102 (16)
Certified recovery specialist/Community health worker	90 (14)
Community health advisor	55 (8)
Health educator	55 (8)
Health interpreter or translator	37 (6)
Outreach worker	28 (4)
Enrollment coordinator	16 (2)
Patient navigator	15 (2)
Family advocate	13 (2)
Peer counselor	3 (1)
Other title	24 (4)
Did not respond	2 (1)

**Table 4:** Primary Sectors Represented

Sector (N = 648)	No. (%)
Social and community services	155 (23.9)
County health department	97 (15)
Private health sector	85 (13.1)
Civic engagement	80 (12.3)
Managed care organization	58 (9)
Advocacy	29 (4.5)
Public safety and law enforcement	29 (4.5)
Business	26 (4)
Education	17 (2.6)
Federally qualified health center	14 (2.2)
Government	11 (1.7)
Other	9 (1.4)
Did not respond	36 (5.6)

**Table 5:** Demographics of Communities Served

Variables (N = 648)	No. (%)
**Sex**	
Male	386 (60)
Female	228 (35)
Both	22 (3)
Other	6 (1)
Did not respond	6 (1)
**Race/Ethnicity**	
White	285 (43)*
Black or African American	44 (7)
Asian	12 (2)
Hispanic or Latino	12 (2)
Native Hawaiian or Pacific Islander	4 (1)
Two or More Races	288 (44)
Did not respond	3 (1)
**Age**	
Children (younger than 12)	23 (4)
Adolescents (13–18)	151 (23)
Adults (18–65)	409 (62)
Elderly (older than 65)	62 (10)
Did not respond	3 (1)
**Immigration status**	
U.S. born/native	523 (81)
Foreign-born/immigrant	113 (17)
Refugees	8 (1)
Did not respond	4 (1)
**Type of community primarily served**	
Rural	117 (18)
Urban	377 (58)
Suburban	133 (21)
Did not respond	21 (3)

*Due to a classification error in the survey, the category of “American Indian or Alaska Native” is combined here with the category of “White”. Based on US census data, American Indian and Alaska Native accounts for 0.4% of the population of Indiana

### Topic 2: attributes, roles, and responsibilities

CHWs and employers both described key CHW attributes, characteristics, roles, and responsibilities, six of which aligned with the national CHW Core Consensus (C3) Project, which aims to increase visibility and a greater understanding of CHWs [23]. Following a post-hoc qualitative analysis, these nationally recognized C3 qualities, roles, and skills were mapped to our Topic 2 findings in Table 2. CHWs are often motivated by their *“want to work with a lot of people [from] different backgrounds”* and from the community they serve, or they leverage their own lived experience to connect and relate with clients. CHWs view themselves as trusted members of the community who actively work to create and keep this trust, *“…it’s usually just a matter of them [clients] getting to know and trust you.”* CHWs acknowledged that trust could be difficult to build but described qualities they possess that allow them to effectively develop this between themselves and their clients such as having patience, the ability to create a safe space, and the need for *“compassion and passion for what you are doing.”* Additionally, CHWs understand the importance of *“meeting [clients] where they are”* and of being creative and flexible. Being adaptable also helps CHWs overcome obstacles when carrying out their work as they, *“come up against… a lot of barriers, and you’ve got to be very creative at how you address… those issues. You don’t want to burn any bridges.”*

A key CHW role is the ability to *“serve as a resource for people that I come in contact with.”* As such, they often become a point person for their clients or *“that one consistent face and voice that [clients] could contact.”* They view *“knowing the resources that we have in the community [as] very important”* as it strengthens their capacity to connect clients to needed medical and social services. As trusted resources, CHWs often advocate for change, *“I’m a health advocate, here at the hospital we have all kinds of projects. Our goal is to break those barriers [experiences by clients, such as] why people sit in the ER forever, you know, constantly, they can’t afford their medications, utilities…just can’t get to the doctors…the transportation.”* In addition to linking clients to resources, CHW responsibilities involve providing case management, offering health programming, and working with clients to set health and wellness goals. Survey results revealed that CHWs work within a wide breadth of health-related topics with chronic disease management being a key focus (13%) (Table 6).

**Table 6:** Primary Focus Area

Focus Area (N = 648)	No. (%)
Chronic disease management	81 (12.5)
Behavioral/mental health	69 (10.6)
General health systems navigation	47(7.3)
Adolescent health	40 (6.2)
Environmental and occupational health	37 (5.7)
Social service eligibility screening	33 (5.1)
Substance abuse	29 (4.5)
Men’s health	23 (3.5)
Emergency response	22 (3.4)
Asthma	22 (3.4)
Communicable diseases (not HIV/AIDS)	20 (3.1)
Dental health	19 (2.9)
Elder health	18 (2.8)
HIV/AIDS	18 (2.8)
Domestic violence	16 (2.5)
Enrollment	16 (2.5)
Cancer	15 (2.3)
Prevention (nutrition/physical activity)	15 (2.3)
Community violence	14 (2.2)
Maternal and child health	14 (2.2)
Injury prevention	14 (2.2)
Smoking and tobacco use	13 (2)
Women’s health	12 (1.9)
Lead poisoning	8 (1.2)
End of life	8 (1.2)
LGBTQ+ issues	6 (0.9)
Tuberculosis	2 (0.3)
Veterans’ services	2 (0.3)
Other	14 (2.2)
Did not respond	3 (0.5)

### Topic 3: recruitment and hiring

Employers shared ways that hired CHWs enhanced their organizational work as *“there was so much that our registered nurses and dietitians and registered respiratory therapists couldn’t complete…so CHWs were hired to help us close those gaps.”* When hiring future CHWs, employers looked for professionalism, flexibility, and organizational and communication skills. Employers also expressed a preference for bilingual candidates with an existing connection to the focus community, *“We really try to hire from within that community. So we’re getting people who know the community, they know the resources, they know people coming into the clinic.”* During the hiring process, employers reported turning to organizational leadership, community partners, and currently employed CHWs. Successful CHW hiring strategies included drafting clear job descriptions, recruiting directly from client populations, hiring CHW cohorts for group training and onboarding, and providing employee benefits.

### Topic 4: certification and training

After analysis, among the CHWs surveyed, over 80% (*n* = 526) reported being certified, more than 50% (*n* = 368) found CHW employment as a result of certification, 12% (*n* = 75) had a CHW position before becoming certified, 5% (*n* = 35) did not find CHW employment, and 6% (*n* = 40) did not seek CHW employment. Participants shared that certification provided CHW work with legitimacy and validation, *“being certified as a CHW, just kind of gives us that little bit of …, I guess, upper level, you know what I mean?”* Certification also seemed to offer CHWs an opportunity to strengthen overall skills, knowledge, and training, *“…it’s just good to have that [training] knowledge and background and experience to add to what we do every day.”*

Surveyed CHWs shared an unawareness of certification when they entered the workforce but were asked to complete certification by their employer. Most (*n* = 492; 76%) CHWs reported employers paying for the CHW certification; employers use state-funded training and workforce development reimbursement programs to do so. However, employers shared inconsistencies in terms of sponsors, certification type (e.g., combined CHW/Certified Recovery Specialist [CRS], stand-alone CHW certification), and cost, *“… it [certification] was kind of expensive… it was even more money than just doing the whole CRS/CHW program. So I think that could deter some people from becoming CHWs.”*

Both CHWs and employers expressed being confused by the different certification programs as some are recognized by the certifying state agency while others are not. They also shared frustration over the lack of clear guidance related to which certification—CHW versus CHW/CRS—is approved for reimbursement. Participants expressed a strong preference for in-person trainings over online versions as it provided an opportunity to connect and network with other CHWs and *“…to help with engaging and just the motivational interviewing skills. I mean, there’s some things that you can’t learn by using the computer and doing virtual.”* While most agreed, some described the required week-long training as quite intensive and suggested that the in-person training be extended or spread across a longer time-period.

### Topic 5: job satisfaction and challenges

Making a difference in people’s lives was described as a rewarding aspect of CHW work when “y*ou can see how you affected them or how, you know, you’re even able to make them believe in themselves, which is huge.”* Being recognized by the medical team was also seen as rewarding: *“I feel like the medical team really recognize after, you know, they definitely are thankful and appreciative of the help that we provide.”* Despite the rewards, CHWs continue to face challenges due to a general lack of awareness of who the CHW workforce is and what they do, which *“...a lot of the time with CHWs, I think that what we do is not valued. So we don’t really get paid that amount that we should get paid.”* CHWs also described barriers such as a lack of resources to help the vulnerable people they serve, which can exact heavy physical and emotional costs. CHWs discussed ways to address these challenges including creating *“more acceptance of what we do and being more respected as a profession because this is a profession.”*

### Topic 6: measuring impact

Both CHWs and employers identified a need to document the impact of CHW work because *“…you help that one person today, you did just a little something for somebody here and there. Over time, that’s going to add up to big success.”* CHWs expressed wanting to be involved in defining and measuring this impact and suggested that metrics to include (e.g.; established community relationships, resource provision to clients, programmatic benchmarks, enrollment goals). Employers agreed a need exists to document CHW impact that supports return-on-investment (ROI) to justify current and future CHW hiring lines. Employers shared various client- and program-level metrics used to track CHW efforts (e.g.; community outreach events conducted, completed screening tools, number of goals set/achieved with clients, changes in knowledge gains, follow-up rates). Employers also discussed community-specific indicators, such as emergency room utilization rates, changes in public safety calls, number of jobs created, and greenspaces developed/improved. Additionally, they explained that metrics from existing registries and assessments are used, such as electronic medical record (EMR)-based registries, Recovery Inventory, Star, Strength and Risk Assessment, Child and Adolescent Needs and Strengths and Adult Needs and Strengths Assessment (CANS/ANSA) state tools, and Insight Vision. Despite these existing approaches, employers agreed that efforts are needed to standardize current tools, reporting systems need to align with existing EMR systems, measures are needed across the social determinants of health, and systems created to build evaluation capacity.

### Topic 7: supervision, professional development, and peer support

Being relatable, supportive, and familiar with CHW work were identified as characteristics of an ideal supervisor, *“I need a supervisor who does this job... And I want someone who has done the work to be able to ‘train up’ others and also be that lead...”* Related to professional development, CHWs expressed wanting access to supervisor encouraged and funded trainings and certifications that go beyond initial CHW certification, but shared that professional development opportunities are not readily available and costs fall upon the CHWs themselves. CHWs shared interest in on-the-job training in particular areas (e.g., home visits, and going into homes), and access to annual conference or annual/quarterly trainings in order to stay current in the field and learn from peers. CHWs also expressed concern over lack of career advancement pathways, *“so within the organization, there are not too many [promotions].”* Despite the absence of professional advancement, CHWs expressed a desire to expand their current position to include supervision and training of other CHWs, *“I would like to teach the classes as well as even after going through the course, inquired about how to become an instructor.”*

CHWs expressed a strong interest in connecting with other CHWs in person at their workplace and through annual conferences. They described INCHWA as playing a key role in providing CHW peer support by engaging CHWs via email, huddles, and webinars, *“I do find the INCHWA emails and newsletters to be helpful…that’s really the only connection that I have to anyone else.”* They also felt that INCHWA’s efforts to advocate for the profession were needed and appreciated.

### Topic 8: funding and reimbursement

CHW positions are primarily funded through county, state, and federal grants or soft-monies and, to a lesser degree, by institutional or hard-money leading to concerns related to job security, *“We work for a not for profit, so things change all the time. Funding could go at any moment...”* Both employers and CHWs shared how soft-money funded programs place employees at risk for unexpected terminations: *“…their [CHW] grant was from the Office of Minority Health, they really hated it because it got pulled in year three and they had made so much progress…I’m staying away from grants because you can’t trust them. The work they did was so valuable…so it was really sad they couldn’t finish.”*

Participants expressed confusion and lacked understanding related to CHW billing for Medicaid reimbursement. They shared concern regarding the sustainability of a business model that relies on the current CHW reimbursement structure given that it does not allow for billing of clients with a diverse payer mix, does not provide payment for CHW services provided on the same day as other provider services, and fails to cover a broad spectrum of CHW services, *“But I think that insurance companies like Medicaid and SSI would benefit in the long run, if they covered more of the services CHWs do because they’re all services that keep their patients, their customers in compliance.”* Both CHWs and employers agreed that applying for CHW reimbursement through Medicaid is cumbersome, confusing, and complex, *“…it would be nice to see more guidance on what’s considered appropriate within those ranges…it’s hard to get a concrete understanding of what’s a part of that CHW service definition.”* Employers discussed the potential of Z code usage, and that more effective collaboration among doctors, providers, and CHWs would be needed to address these challenges, *“…if our doctors or providers would work closer together, and they would look through the curriculum with me, we could work as a partner, and I could educate, educate the group and get reimbursed for his patients.”*

## Discussion

**Fig. 1:**
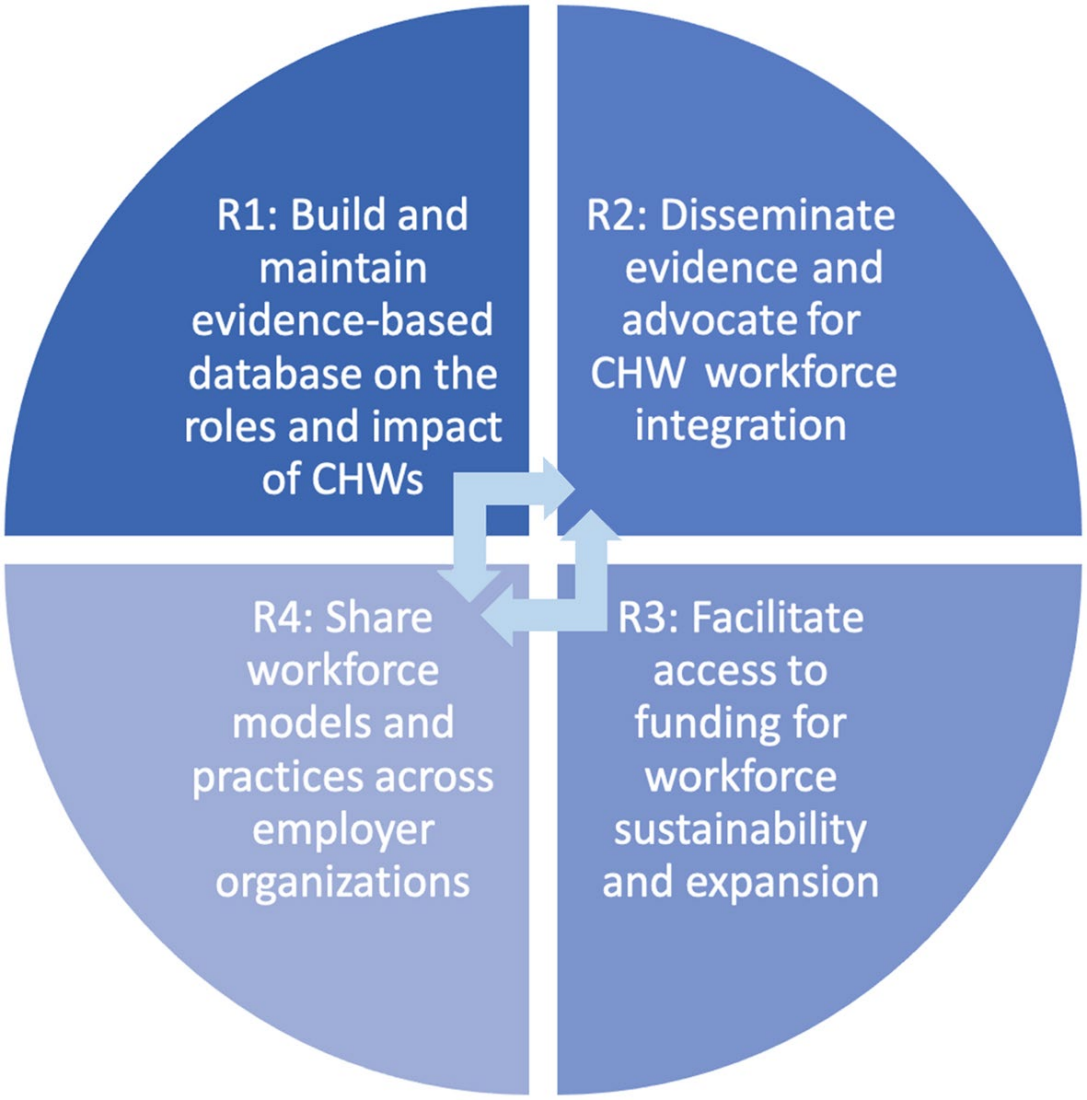
Key recommendations for expanding and sustaining Indiana’s CHW workforce

With growing evidence of the clinical and social impact of CHWs in communities that are underserved, there is an increased national demand for CHWs. Despite this, some US states continue to face challenges related to funding, training, expanding, and sustaining their CHW workforce [24]. Engaging key stakeholders from multiple perspectives and utilizing a multi-methods assessment approach provided invaluable insight into the strengths, needs, and landscape of the CHW workforce in Indiana. These collaborative efforts positioned us to prioritize actionable recommendations. Research findings demonstrated the strengths and diversity of Indiana CHWs and informed the authors’ four key recommendations for expanding and sustaining Indiana’s CHW workforce. These recommendations are transferable to other states with similar contexts and can be implemented sequentially or concurrently with no prescribed order (Fig. 1).

### Recommendation 1: standardize, build, and maintain a database on the roles and impact of CHWs in Indiana

Scaling and sustaining CHW positions is best facilitated through accessible information documenting the roles, responsibilities, and impact of CHWs. Clearly defining CHW roles to healthcare providers allows for better CHW integration into healthcare organizations, improving overall health outcomes [6, 25]. Maintaining an Indiana CHW registry will allow compilation of up-to-date statistics on workforce density, geography, certification levels, areas of work, employment, and income. With standardized data collection across employer organizations, it is possible to measure the full impact of CHW services and demonstrate ROI, as well as establish standardized and sector-specific metrics for CHW outputs and outcomes. Ingram et al. [4] described the use of electronic health records to examine the effect of CHW services on chronic disease outcomes. The Arizona Community Health Worker Association and the CHW Workforce Coalition spent 2 years gathering data from and about CHWs and community health representatives serving American Indian Tribes and communities via small group sessions and annual meetings. These data later informed a formalized assessment of CHW mastery of core competencies as well as a process to approve training programs throughout the state [26]. Our recommendation to build and maintain an up-to-date evidence based CHW registry and database could serve as a more efficient approach to collecting data, and meaningfully build upon the mounting evidence of positive health outcomes associated with the integration of CHWs into patient care across the country [3–5, 27–32]. These standardized data can be used to inform and catalyze advocacy and policy efforts towards creating and/or refining standardized state training programs, pathways for certification, employment, and sustainable funding for the CHW workforce.

### Recommendation 2: disseminate evidence and educate employers and communities about the CHW profession to advocate for CHW workforce integration and expansion

Dissemination of evidence can be more easily achieved upon establishment of the evidence-based database (Recommendation 1), which could inform the development of dissemination products, such as informational materials and strategies, and can equip all applicable stakeholders with increased clarity and understanding of the CHW profession, associated roles, and impact on health outcomes. The National Association of Community Health Workers (NACHW) document resource center is one example of data dissemination, which is accessible on the NACHW website for free and includes study reports, policy briefs, fact sheets, and more from researchers and CHW organizations across the country [33]. In accordance with Sabo et al. [24], we recommend that evidence be distributed in lay language and accessible through a variety of outlets (e.g., social media, newsletters, workshops, interactive presentations at staff meetings), in the preferred language of the focus community. Communities who understand the role of CHWs will be more trusting of the profession and are better poised to leverage CHW services. Employers who know the demands of the job are also in a better position to take the steps to adequately support the workforce [34]. Yearly reviews and pre- and post-program implementation analyses are vital in promoting workforce development. Frequent data analysis and dissemination of organizational-level CHW impact metrics will support evidence-based advocacy for the workforce. Impact metrics can include the number of enrolled patients, client evaluation forms, follow-up rates, and community-specific indicators such as a reduction in emergency room use [2]. The C3 Project, which aims to increase visibility and a greater understanding of CHWs, created a publicly available CHW assessment toolkit. The toolkit allows for field-oriented, evidence-based recommendations and resources that aid in the holistic assessment of CHW impact [23]. Similarly, our recommendation supports creating and disseminating informational products that are readily accessible and can demonstrate the positive impact that CHWs have on their communities, thus, leading to opportunities for increased funding, expanded policy initiatives and resources for employing and retaining CHWs.

### Recommendation 3: facilitate understanding and promote access to and utilization of existing funding mechanisms for CHW workforce sustainability and expansion

An underutilized and misunderstood billing framework for reimbursement of CHW services leads to a lack of sustainable funding for permanent CHW positions. Inconsistent funding jeopardizes CHW recruitment and retention, as well as program implementation. Ensuring sufficient funds through extensive cost analyses that consider expenses associated with continued training, sufficient supervision, and integration of CHWs into healthcare systems is vital [34]. There are various CHW funding strategies to be explored, including reimbursement for non-clinic based CHWs, and leveraging existing state workforce development programs. Our study findings suggest that the existing Medicaid reimbursement policies in Indiana require improved clarity and employer education to increase appropriate utilization [2]. Ibe et al. [35] concluded that more research needs to be conducted to gain a better understanding of CHW certification and its’ impact on patient health outcomes. Similarly, further research on the role of CHW certification in supporting the financial sustainability and workforce development of CHWs in the US is warranted.

A well-maintained evidenced-based CHW database (Recommendation 1) could facilitate advocacy efforts regarding reimbursement rates to be commensurate with the impact of CHWs and better alignment with similar position policies (i.e., Certified Recovery Specialists). Furthermore, the current public health climate due to the Coronavirus Disease 2019 (COVID-19) pandemic has generated additional federal funding mechanisms for CHW career paths. A more recent avenue of federal funding is the increased public health workforce funding through the Centers for Disease Control and Prevention (CDC). As of March 2021, the CDC planned to provide $300 million towards CHW services to support COVID-19 control and prevention and an additional $32 million towards training, technical assistance, and evaluation of CHWs [36]. President Biden’s plan included increasing the CHW workforce by 150,000 nationally and pledged to provide direct grant funding, as well as add CHW services as an optional benefit for states through Medicaid [37]. States can better maximize the sustainability of the workforce as funding opportunities arise to revise existing regulations around CHW reimbursement.

### Recommendation 4: share CHW workforce models and best practices across employer organizations to optimize strategies

Fragmented CHW employment pathways and lack of career development opportunities can lead to missed opportunities, stagnant wages, job dissatisfaction, and high turnover rates. These can be mitigated by the creation of standardized, sector-specific guidance for optimal recruitment, integration, training, and ongoing education, certification, supervision, career development, and funding of CHWs for employers in Indiana. The National Committee for Quality Assurance (NCQA), in partnership with the Penn Center for Community Health Workers, is a multi-stakeholder effort to create such standards nationally. The goal of the NCQA standards is less about dictating CHW training methods or scope of practice and more about providing evidence-governed guidelines for the systems in which CHWs work [38]. Involving CHWs at all levels of decision-making related to the workforce will streamline communication across CHW employer organizations, state agency partners, and other stakeholders to align state-wide efforts. Yearly reviews of national CHW models and standards should be conducted to remain aligned with best practices outside of Indiana and coordinate with efforts already underway (e.g., APHA–CHW section, NACHW). Demonstration projects can allow for the development of context-specific guidance to help integrate CHWs and scale up workforce models into organizational strategic plans, teams, and workflows across the state.

### Limitations

There were several limitations to this study. While our steering committee guided our work, participants of the focus groups did not capture all potential stakeholders (e.g., unemployed CHWs, policy makers, and national organizations who work with CHWs). A disproportionate percentage of CHWs selected “American Indian or Alaska Native” as their race/ethnicity which is inconsistent with the known demographics of Indiana. Future surveys should be created to more clearly delineate the race/ethnicity of all respondents in order to avoid this classification error. Methods taken to eliminate potentially duplicative and fraudulent survey responses as described above may have inadvertently eliminated legitimate responses. Future online surveys should utilize software features, such as prevention of “ballot stuffing”. Lastly, the findings presented here are specific to Indiana and may not be generalizable to all states; thus, each state should identify unique nuances of the CHW workforce in their geographic region.

## Conclusion

CHWs come from diverse cultural and linguistic backgrounds, with an existing connection and relatability to the focus community—valued attributes and characteristics by employers. Through their knowledge base, skills, and social networks, CHWs excel at serving as an essential conduit for locating and connecting clients with both social and medical resources which leads to reduced health disparities and improved individual and community health outcomes. A high need for CHWs exists across community and clinical settings; however, employers often lack the ability to obtain and sustain proper funding to meet this demand. Despite a growing CHW workforce, a consistent foundation and understanding of CHW positions and capabilities is lacking. Creating consistency and a greater understanding regarding CHW models, measures, evidence-based practices, funding and reimbursement policies, and training and certification is crucial to optimize strategies and build workforce capacity locally and nationally.

## Data Availability

Complete data set is available for review upon request. Please contact the first author Dr. Natalia Rodriguez to request data from this study. Email: natalia@purdue.edu

## Appendix

### Appendix A: Employer and CHW Focus Group Guides

**INTRODUCTION**

1. How many CHWs does your organization currently employ?

- How many would you ideally like to employ/have capacity for?
- Do you have imminent needs/plans in place to hire more CHWs?

2. How and from where do you/would you recruit your org’s CHWs?

- Are CHWs hired to work within their own community?

3. What do the CHWs employed by your organization do?

- Are there specific tasks and why were CHWs specifically hired to do that? (As opposed to social workers, etc.…)

**IMPACT/OUTCOMES**

1. What strengths do the CHWs bring to your org?
2. What data are you collecting to measure their impact/success?
3. On a scale of 1–5, how important is it for your organization to prioritize the expansion of CHWs?

**BARRIERS TO CHW EMPLOYMENT/SUSTAINABILITY**

4. How are you currently financially supporting CHWs within your organization?

- Has that changed?
- Is it only based on grants? Is it for specific projects? What happens when projects end?
- Are any purely volunteers?

5. In what ways have you had to justify CHW positions in your budget?

- Besides above, is there additional specific data you need to collect for your grants?

6. Do you currently utilize CHW education codes for Medicaid reimbursement?

- If no:

i. Why not? What are the challenges? Have you heard of this?
ii. If you could propose a new reimbursement model, what would it look like?

b. If yes:

i. Please describe the process of billing and reimbursement.
ii. What’s working?
iii. What’s not?
iv. Does the reimbursement sustain the entire CHW position or are other sources of funding needed?
v. If you could propose a new reimbursement model, what would it look like?

7. Think about when you first created a CHW position. What are the barriers you faced for implementing this position?

- Thinking about your current CHW model/positions, what are the barriers you face for sustainability and/or expansion?

i. How do you identify the next population/community to expand to?

8. What resources do you need to implement or sustain CHW positions?

- What kinds of support/trainings do you as employers need? *(What kind of support could you use in order to maintain a budget line for your CHWs or bring in new funding for new projects/expansion)*
- What kinds of support/trainings do your CHWs need?

9. Who is currently training your CHWs?

- Is certification required for employment? Whose responsibility is it to pay for certification?
- Are they trained for specific projects?
- Do you offer/support CE or additional training opportunities?

**SUPERVISION**

10. What do supervision models look like for CHWs?
11. What do career development models look like for CHWs?

- Are there clear pathways for promotion?

**What else would you like us to know about CHWs within your organization?**

CHW Focus Group Guide

**INTRODUCTION**

1. What led you to become a CHW?
2. What are the main goals of your job?
3. If you could change anything about your CHW responsibilities, what would it be?
  - Is there more you would like to do to support the clients you work with?
4. What makes you a good CHW? What strengths/skills do you bring to your position?

JOB SATISFACTION/WHAT MAKES GOOD JOB

5. What do you find most rewarding about your job?
  - Do you feel recognized and respected in your position?
  - What is the potential for growth in your position?
  - Would you refer friends and family members to becoming a CHW and working in this field? Why or why not?
6. Do you feel you are compensated fairly in your current position?
7. What is the #1 challenge of your job?
8. What resources do you need to do your job more effectively/efficiently?
9. How does your employer define success of your work/position?
  - If you wanted to convince your employer to hire more CHWs, what would they have to see?

COMMUNITIES THEY SERVE/ PROBLEMS THEY’RE ADDRESSING

10. Tell us about your community that you work in. What are the biggest issues that are faced?
11. How do you define success of your work/position within the community you’re serving?

EMPLOYER SUPERVISION/SUPPORT

12. Do you feel that you have job security in your current position?
  - Do you feel your position is time-limited?
  - What do you know about how your position is funded?

PERSPECTIVES ON CERTIFICATION AND TRAINING

13. Think about the trainings you’ve received as a CHW. What additional trainings do you feel you need?
  - What is your interest in ongoing training, renewal, continuing education?
14. What opportunities do you have for ongoing training?
  - Do they come from your employer?
  - From outside sources?
15. What are your thoughts on CHW certification?
  - What was the process like?
  - What would you change about the process?
  - Any challenges to obtaining certification?
  - Does it lead to higher wages or more/better employment opportunities? Is it worth it?

**PEER SUPPORT**

16. How do you connect with other CHWs within or outside of your organization?
  - How do you engage with INCHWA? How does it meet your needs as a prof. Organization? What more would you like to see from INCHWA?
  - In what other ways would you like to connect with CHW peers?

PROFESSIONAL DEVELOPMENT

17. What, if anything, would you like to do as the next stage of your career?
  - What, if any, aspirations do you have beyond CHW work?

**Is there anything we should have asked but didn’t?**

**Can we contact you in the future if we have follow-up questions?**

## Abbreviations

CHW: Community Health Worker
CHWDI: Community Health Worker Development Institute
NASPH: National Academy for State Health Policy
IRB: Institutional Review Board
INCHWA: Indiana Community Health Worker Association
ROI: Return-on-Investment
EMR: Electronic Medical Record
CANS: Child and Adolescent Needs and Strengths
ANSA: Adult Needs and Strengths Assessment
NACHW: National Association of Community Health Workers
C3: CHW Core Consensus Project
CDC: Center for Disease Control and Prevention
NCQA: National Committee for Quality Assurance
